# Semi‐parametric analysis of overdispersed count and metric data with varying follow‐up times: Asymptotic theory and small sample approximations

**DOI:** 10.1002/bimj.201800027

**Published:** 2018-12-05

**Authors:** Frank Konietschke, Tim Friede, Markus Pauly

**Affiliations:** ^1^ Department of Mathematical Sciences University of Texas at Dallas Dallas TX USA; ^2^ Department of Medical Statistics University Medical Center Göttingen Göttingen Germany; ^3^ Institute of Statistics Ulm University Ulm Germany

**Keywords:** permutation methods, resampling, studentized statistics

## Abstract

Count data are common endpoints in clinical trials, for example magnetic resonance imaging lesion counts in multiple sclerosis. They often exhibit high levels of overdispersion, that is variances are larger than the means. Inference is regularly based on negative binomial regression along with maximum‐likelihood estimators. Although this approach can account for heterogeneity it postulates a common overdispersion parameter across groups. Such parametric assumptions are usually difficult to verify, especially in small trials. Therefore, novel procedures that are based on asymptotic results for newly developed rate and variance estimators are proposed in a general framework. Moreover, in case of small samples the procedures are carried out using permutation techniques. Here, the usual assumption of exchangeability under the null hypothesis is not met due to varying follow‐up times and unequal overdispersion parameters. This problem is solved by the use of studentized permutations leading to valid inference methods for situations with (i) varying follow‐up times, (ii) different overdispersion parameters, and (iii) small sample sizes.

## 1 INTRODUCTION

Metric data and especially count data are common endpoints in clinical trials. Examples include relapses and magnetic resonance imaging (MRI) lesion counts in relapsing‐remitting multiple sclerosis (MS), exacerbations in chronic obstructive pulmonary disease (COPD), and hospitalizations in heart failure. For several of these the negative binomial distribution has been suggested to be an appropriate model accounting for between‐patient heterogeneity in event rates manifesting in overdispersion, that is variances exceeding the means. For instance, Wang, Meyerson, Tang, and Qian (2009) suggested the negative binomial model for the analyses of relapses, and Sormani et al. (1999, 2001, 2005) and Van den Elskamp, Knol, Uitdehaag, and Barkhof (2009) for various types of MRI lesion counts in MS. Based on two large‐scale COPD trials, Keene, Calverley, Jones, Vestbo, and Anderson (2008) assessed various models and recommended the negative binomial model for application. In the situations described above, commonly analyses methods (e.g. PROC GENMOD in SAS) are applied based on large sample properties of underlying Maximum‐Likelihood‐Estimates (MLE) and the assumption of a common overdispersion parameter across treatment groups. Such distributional assumptions, however, can hardly be verified; especially in case of small to moderate sample sizes (Aban, Cutter, & Mavinga, 2009). Even if the distribution is correctly specified the MLEs of the overdispersion parameters are biased (Link & Sauer, 1997; Lord, 2006; Paul & Islam, 1995; Saha, 2011; Saha & Paul, 2005) that may lead to wrong conclusions. Moreover, it is quite common that varying follow‐up times occur, see for example, Chen et al. (2013), McCullagh and Nelder (1989). All of the above‐ mentioned characteristics may not only be shared by count data, but also by metric data measured on an arbitrary scale. Simultaneously accommodating all of these complications in an accurate statistical inference method in a unified way is a rather challenging task. To the best of our knowledge no suitable methods currently exist that can simultaneously handle heteroscedastic data (counts) with varying follow‐up times.

It is the aim of the present paper to develop valid inference procedures for the analysis of such data in general models allowing for possibly time‐varying follow‐up times and different overdispersion parameters in a nonparametric way. This is accomplished by newly derived unbiased estimators (based on the methods of moments) for the (count) rates and their variances. The rigorous study of their large sample properties then leads to asymptotically correct tests and confidence intervals for treatment effects using critical values from the standard normal distribution.

With small samples the use of normal quantiles for inference can lead to liberal or conservative decisions whereas permutation tests offer an opportunity to derive quantiles from appropriate reference distributions. In particular, the application of studentized permutation procedures is tempting since they have been shown to control the type‐*I*‐error rate very accurately in various situations (Chung & Romano, 2013; Chung & Romano, 2016; Janssen, 1997; Konietschke & Pauly, 2014; Pauly, Brunner, & Konietschke, 2015). The problem in this particular situation is that with varying follow‐up times and unequal overdispersion parameters the usual assumption of independently identically distributed (*iid*) observations in the groups is not met. This issue can be solved by applying more general theorems on permutation statistics by Janssen and Pauls (2003) and Janssen (2005) and Pauly (2011). Even though data may not be exchangeable under the null hypothesis, the derived permutation methods are asymptotically correct in that they control the type I error rate or the coverage probability for hypothesis tests and confidence intervals, respectively.

The paper is organized as follows: The statistical model and point estimates are given in Section 2. Unbiased variance estimators are provided in Section 3. In Section 4, test procedures and confidence intervals are derived. Permutation‐based small sample size approximations and simulation results are presented in Section 5. Finally, two illustrative data examples are analyzed in Section 6. The paper closes with a discussion of the proposed methods in Section 7. All proofs are given in the supplement to this paper.

## 2 STATISTICAL MODEL, POINT ESTIMATES, AND MULTIVARIATE NORMALITY

We consider a general semi‐parametric two‐sample layout with independent random variables $X_{ik}$ with

(1) $$\begin{array}{c} E\left(X_{ik}\right)=t_{ik}\lambda_{i}\;\;and\;\;Var\left(X_{ik}\right)=\sigma_{ik}^{2},\;i=1,2,\;k=1,\ldots ,n_{i}. \end{array}$$

Here, the index *i* represents the treatment groups (i=1 control, and i=2 treatment), and *k* the subject within treatment group *i* with individual follow‐up time $t_{ik}$, and $\lambda_{i}>0$ the expectation of group *i*. Note that the variance $\sigma_{ik}^{2}$ may depend on $t_{ik}$, for example if $X_{ik}$ follows a Negative Binomial distribution (in this special case $\sigma_{ik}^{2}=t_{ik}\lambda_{i}+t_{ik}^{2}\lambda_{i}^{2}\phi_{i}$), a Poisson distribution ($\sigma_{ik}^{2}=t_{ik}\lambda_{i}$), or an Exponential distribution (here $\sigma_{ik}^{2}=t_{ik}^{2}\lambda_{i}^{2}$). We further assume that the fourth moments exist and are bounded, that is $\sup_{k\geq 1}E\left(X_{ik}^{4}\right)\leq C_{0}<\infty$ for a constant $C_{0}>0$ and i=1,2.

The design is allowed to be completely heteroscedastic, that is every observation might have a different expectation and variance. All statistical procedures for the analysis of *iid* observations are inappropriate for statistical inference in model (1). Let $N=\sum_{i=1}^{2}n_{i}$ denote the total sample size, $T_{i}=\sum_{k=1}^{n_{i}}t_{ik}$ the total follow‐up times in group *i*, i=1,2, and let $T=\sum_{i=1}^{2}T_{i}$ denote the total follow‐up times across both treatment groups. The unknown rate parameters $\lambda_{i}$ can be estimated without bias by

(2) $$\begin{array}{c} {\hat{\lambda }}_{i}=\frac{1}{T_{i}}\sum_{k=1}^{n_{i}}X_{ik} \end{array}$$

and can be interpreted as a weighted mean of the data. The variance of ${\hat{\lambda }}_{i}$ is given by

(3) $$\begin{array}{ccc} \sigma_{i}^{2} & = & Var\left({\hat{\lambda }}_{i}\right)=\frac{1}{T_{i}^{2}}\sum_{k=1}^{n_{i}}\sigma_{ik}^{2}. \end{array}$$

For the derivation of asymptotic results for the rate estimates (2), the following mild regularity conditions on sample sizes and follow‐up times are required:

(4) $$\begin{array}{ccc} & & t_{ik}\in \left[L,U\right]\;where\;0<L<U<\infty , \end{array}$$

(5) $$\begin{array}{ccc} & & N\to \infty \;such\;that\;\frac{n_{i}}{N}\to \kappa_{i}\in \left(0,1\right), \end{array}$$

(6) $$\begin{array}{ccc} & & T\to \infty \;such\;that\;\frac{T_{i}}{T}\to {\tilde{\kappa }}_{i}\in \left(0,1\right), \end{array}$$

(7) $$\begin{array}{ccc} & & \frac{1}{T_{i}}\sum_{k=1}^{n_{i}}\sigma_{ik}^{2}\to {\tilde{\tau }}_{i}^{2}\in \left(0,\infty \right),\;as\;T_{i}\to \infty . \end{array}$$

Assumption (4) ensures that the follow‐up times appear on a fixed time interval of interest, while Assumptions (5)–(7) guarantee the existence of limiting variances of the point estimates, see Theorem 2.1 below. In particular, it follows immediately, that the estimator ${\hat{\lambda }}_{i}$ is consistent as $n_{i}\to \infty$ and $T_{i}\to \infty$, respectively. However, the variance $\sigma_{i}^{2}$ defined in (3) represents an unknown weighted sequence of the quantities $\sigma_{ik}^{2}$, which depends on both the follow‐up times and sample sizes. Thus, it cannot be represented by model constants. In order to derive inference methods for the general hypothesis $H_{0}:h\left(\lambda_{1},\lambda_{2}\right)=\theta_{0}$, however, the estimator needs to be multiplied by adequate known coefficients, such that $\sigma_{i}^{2}$ converges to a specific variance constant, which is, asymptotically, unaffected by the follow‐up times and sample sizes. The result along with the multivariate normality of the estimator $\hat{\lambda }={\left({\hat{\lambda }}_{1},{\hat{\lambda }}_{2}\right)}^{\prime }$ of $\lambda ={\left(\lambda_{1},\lambda_{2}\right)}^{\prime }$ are given in the next theorem.

Theorem 2.1

(1) Under Assumptions (4), (6), and (7),
  (8) $$\begin{array}{c} \sqrt{\frac{T_{1}T_{2}}{T}}\left(\hat{\lambda }-\lambda \right)\overset{D}{\to }N\left(0,\Sigma \right),\;where\;\Sigma =diag\left\{{\tilde{\kappa }}_{2}{\tilde{\tau }}_{1}^{2},{\tilde{\kappa }}_{1}{\tilde{\tau }}_{2}^{2}\right\} \end{array}$$
  is a diagonal limiting covariance matrix.

Note that the diagonal covariance matrix Σ neither depends on the sample sizes $n_{i}$, nor on the time‐varying coefficients $t_{ik}$. The matrix, is, however, unknown in practical applications, and needs to be estimated. An unbiased and $L_{2}$‐consistent estimator is derived in the next section.

## 3 ESTIMATION OF THE VARIANCE

Moment‐based estimators for variances denote, roughly speaking, the squared deviation from the mean. In model (1), however, no uniquely defined mean exists. In particular, the variance $\sigma_{i}^{2}$ is a sum of variances, and is not defined as a fixed variance constant. Therefore, the usual sample variance moment‐based estimator is biased, a rather inappropriate characteristic of a variance estimator. Below, we derive an unbiased and consistent moment‐based estimator of $\sigma_{i}^{2}$.

Define the random variables ${\tilde{Z}}_{ik}=X_{ik}-t_{ik}{\hat{\lambda }}_{i}$, and note that $E({\tilde{Z}}_{ik})=0$ for all i=1,2, and $k=1,\ldots ,n_{i}$. The variables ${\tilde{Z}}_{ik}$ describe the deviation of $X_{ik}$ to its estimated expectation. An unbiased moment‐based estimator can now be derived by considering the squared deviation from ${\tilde{Z}}_{ik}$ along with a bias correction. Define

(9) $$\begin{array}{c} K_{i}=\sum_{k=1}^{n_{i}}\frac{t_{ik}^{2}}{\left(T_{i}-2t_{ik}\right)T_{i}} \end{array}$$

and consider

(10) $$\begin{array}{c} {\tilde{\sigma }}_{i}^{2}=\frac{1}{\left(1+K_{i}\right)T_{i}^{2}}\sum_{k=1}^{n_{i}}\frac{T_{i}}{\left(T_{i}-2t_{ik}\right)}{\tilde{Z}}_{ik}^{2}. \end{array}$$

The estimator ${\tilde{\sigma }}_{i}^{2}$ is not a usual sample variance estimator, since it only involves sums of the follow‐up times $t_{ik}$ as weighting factors. However, it describes the mean squared deviation from the observations $X_{ik}$ to their estimated mean $t_{ik}{\hat{\lambda }}_{i}$. Further let

(11) $$\begin{array}{c} \hat{\Sigma }=diag\left({\hat{\sigma }}_{1}^{2},{\hat{\sigma }}_{2}^{2}\right)=\frac{T_{1}T_{2}}{T}diag\left({\tilde{\sigma }}_{1}^{2},{\tilde{\sigma }}_{2}^{2}\right) \end{array}$$

denote the diagonal matrix with diagonal elements $\frac{T_{1}T_{2}}{T}{\tilde{\sigma }}_{1}^{2}$ and $\frac{T_{1}T_{2}}{T}{\tilde{\sigma }}_{2}^{2}$, respectively. It is shown in the next theorem, that ${\tilde{\sigma }}_{i}^{2}$ is an unbiased estimator of $\sigma_{i}^{2}$ and that $\hat{\Sigma }$ is $L_{2}$‐consistent.

Theorem 3.1 For each i=1,2 the estimator ${\tilde{\sigma }}_{i}^{2}$ is an unbiased estimator of $\sigma_{i}^{2}$. Moreover, the estimator $\hat{\Sigma }$ is $L_{2}$‐consistent, that is

$$\left|\right|\hat{\Sigma }\Sigma^{-1}-I_{2}{\left|\right|}_{2}^{2}\to 0,\;T\to \infty .$$

A detailed proof is given in the supplementary material.

We note that the variance estimator ${\tilde{\sigma }}_{i}^{2}$ may become negative in “severe” situations, that is if any $t_{ik}$ is way larger than the others. In this case we suggest to use the asymptotically unbiased version

$$\begin{array}{c} {\tilde{\sigma }}_{i}^{2*}=\frac{1}{n_{i}\left(n_{i}-1\right)}\sum_{k=1}^{n_{i}}{\left(X_{ik}-t_{ik}{\hat{\lambda }}_{i}\right)}^{2} \end{array}$$

of ${\tilde{\sigma }}_{i}^{2}$ instead.

The asymptotic normality of the point estimates and the consistent variance estimates can now be used for the derivation of test procedures and confidence intervals.

## 4 TEST PROCEDURES AND CONFIDENCE INTERVALS

In this section, different procedures for testing the null hypothesis $H_{0}:h\left(\lambda_{1},\lambda_{2}\right)=\theta_{0}$ as well as confidence intervals for the treatment effect $h(\lambda_{1},\lambda_{2})$ will be discussed, where $h:R_{+}^{2}\to R$ is continuously differentiable in $\left(\lambda_{1},\lambda_{2}\right)$. Let $g\left(h\right)=g\left(h,\lambda_{1},\lambda_{2}\right)={\left(\frac{\partial h}{\partial \lambda_{1}},\frac{\partial h}{\partial \lambda_{2}}\right)}^{\prime }$ denote the gradient of *h* with estimator $\hat{g}\left(h\right)=\hat{g}\left(h,{\hat{\lambda }}_{1},{\hat{\lambda }}_{2}\right)={\left(\frac{\partial h}{\partial {\hat{\lambda }}_{1}},\frac{\partial h}{\partial {\hat{\lambda }}_{2}}\right)}^{\prime }$. It follows from the multivariate delta‐method that

(12) $$\begin{array}{c} f\left(h{\left({\hat{\lambda }}_{1},{\hat{\lambda }}_{2}\right)}^{\prime }-h\left(\lambda_{1},\lambda_{2}\right)\right)\overset{D}{\to }N\left(0,\sigma_{h}^{2}\right), \end{array}$$

where

(13) $$\begin{array}{c} f=\sqrt{\frac{T_{1}T_{2}}{T}}\;and\;\sigma_{h}^{2}={\left(g(h)\right)}^{\prime }\Sigma g\left(h\right). \end{array}$$

The variance $\sigma_{h}^{2}$ is unknown, and must be estimated in practical applications. However, $\sigma_{h}^{2}$ is a linear combination of the individual variances $\sigma_{i}^{2}$, respectively. It follows immediately, that a consistent estimator is given by

(14) $$\begin{array}{c} {\hat{\sigma }}_{h}^{2}={\left(\hat{g}\left(h\right)\right)}^{\prime }\hat{\Sigma }\hat{g}\left(h\right). \end{array}$$

Based on the asymptotic normality of $f(h{\left({\hat{\lambda }}_{1},{\hat{\lambda }}_{2}\right)}^{\prime }-h\left(\lambda_{1},\lambda_{2}\right))$ and Slutsky's Theorem, it thus follows that

(15) $$\begin{array}{c} T_{\left(h\right)}\left(\theta \right)=f\frac{\left(h\left({\hat{\lambda }}_{1},{\hat{\lambda }}_{2}\right)-\theta \right)}{{\hat{\sigma }}_{h}}\overset{D}{\to }N\left(0,1\right) \end{array}$$

where $\theta =h(\lambda_{1},\lambda_{2})$. For large sample sizes, the null hypothesis $H_{0}:h\left(\lambda_{1},\lambda_{2}\right)=\theta_{0}$ will be rejected at a two‐sided significance level α, if $|T_{\left(h\right)}\left(\theta_{0}\right)|\geq z_{1-\alpha /2}$, where $z_{1-\alpha /2}$ denotes the (1−α/2)‐quantile of the standard normal distribution. Asymptotic (1−α)‐confidence intervals for θ are obtained from

(16) $$\begin{array}{c} P\left(\theta \in \left[h\left({\hat{\lambda }}_{1},{\hat{\lambda }}_{2}\right)\pm \frac{z_{1-\alpha /2}}{f}{\hat{\sigma }}_{h}\right]\right)\to 1-\alpha . \end{array}$$

## 5 SMALL SAMPLE APPROXIMATIONS AND SIMULATION RESULTS

Extensive simulations were conducted to investigate the accuracies of the test procedures derived in Section 4 for small sample sizes with regard to (i) controlling the type‐1 error rate at the nominal significance level (α=5%), (ii) their powers to detect certain alternatives $H_{1}:h\left(\lambda_{1},\lambda_{2}\right)\neq \theta_{0}$, and (iii) the coverage probabilities of the corresponding confidence intervals in (16). All simulations were conducted with *R* environment, version 2.15.2. (R Development Core Team, 2010), each with $n_{sim}=10,000$ simulation runs.

In all simulations, we focus on testing the hypothesis

(17) $$\begin{array}{ccc} & & H_{0}^{\left(L\right)}:h_{L}\left(\lambda_{1},\lambda_{2}\right)=\log\left(\lambda_{1}/\lambda_{2}\right)=0\;vs.\;H_{1}^{\left(L\right)}:\log\left(\lambda_{1}/\lambda_{2}\right)\neq 0, \end{array}$$

corresponding to the function $h\left(\lambda_{1},\lambda_{2}\right)=L\left(\lambda_{1},\lambda_{2}\right)=\log\left(\lambda_{1}/\lambda_{2}\right)$. The test statistic is given by

(18) $$\begin{array}{ccc} T_{\left(L\right)} & = & f\frac{\log\left({\hat{\lambda }}_{1}/{\hat{\lambda }}_{2}\right)}{\sqrt{{\hat{\sigma }}_{1}^{2}/{\hat{\lambda }}_{1}^{2}+{\hat{\sigma }}_{2}^{2}/{\hat{\lambda }}_{2}^{2}}}, \end{array}$$

which yield to asymptotically valid tests $\psi_{f}=1\{|T_{\left(L\right)}\left|\leq z_{1-\alpha /2}\right\}$ for $H_{0}^{\left(L\right)}$. Moreover, confidence intervals can be derived from (16), respectively. Simulation studies indicate, however, that the statistic $T_{\left(L\right)}$ in (18) tends to result in rather liberal conclusions for small sample sizes ($n_{i}\leq 20$). Therefore, we propose a studentized permutation approach to approximate its sampling distribution for small sample sizes. This will be explained in the next section.

### 5.1 A studentized permutation approach

Permutation tests are widely known to be robust and exact level α tests when the data are exchangeable. Exchangeability implies, however, that variances across the groups are identical. As mentioned above, the data are allowed to be completely heteroscedastic in model (1). Roughly speaking, a usual permutation test would fail to test the null hypotheses formulated above. However, asymptotic permutation tests can be obtained, if appropriate *studentized statistics* are permuted, which will now be briefly explained: It turns out that the test statistic $T_{\left(L\right)}$ follows, asymptotically, a standard normal distribution under the null hypothesis. A permutation or resampling test would now lead to accurate results (at least asymptotically), if the conditional permutation distribution of the test statistic $T_{\left(L\right)}$, say $F^{*}$, would generally mimick the null distribution of the test statistic. That is, both distributions should at least coincide asymptotically. If that is the case, critical values (or *P*‐values) could be computed from the permutation distribution instead of the standard normal distribution for making inferences. Therefore, the goal of the following investigations is to show that the permutation distribution of $T_{\left(L\right)}$, $F^{*}$, is indeed the standard normal distribution. In order to do so, some notations and ideas about the permutation schemes are necessary:

Let $X={\left(X_{11},\ldots ,X_{1n_{1}},X_{21},\ldots ,X_{2n_{2}}\right)}^{\prime }$ denote the pooled sample, and let $t={\left(t_{11},\ldots ,t_{1n_{1}},t_{21},\ldots ,t_{2n_{2}}\right)}^{\prime }$ denote the corresponding vector of the pooled follow‐up times $t_{ik}$. For a fixed, but random permutation π of (1,…,N), let $X^{\pi }={\left(X_{11}^{\pi },\ldots ,X_{1n_{1}}^{\pi },X_{21}^{\pi },\ldots ,X_{2n_{2}}^{\pi }\right)}^{\prime }$ and $t^{\pi }={\left(t_{11}^{\pi },\ldots ,t_{1n_{1}}^{\pi },t_{21}^{\pi },\ldots ,t_{2n_{2}}^{\pi }\right)}^{\prime }$ denote the permuted data and corresponding follow‐up times, respectively.

Permuting X and t using the same random permutation π, the permuted values $X_{ik}^{\pi }$ and $t_{ik}^{\pi }$ are not necessarily independent, which is a rather (at least technically) undesirable property in this context. We therefore propose to permute X and t independently. This is similar to two sample problems with right‐censored survival data, where it is also recommended that the permuted failure times do not occur in general with their corresponding censoring indicators, see Janssen and Mayer (2003) as well as Brendel, Janssen, Meyer, and Pauly (2014). To this end, we consider another random permutation $\pi^{\prime }$ of (1,…,N) that is independent of π and calculate the permuted estimators ${\hat{\lambda }}_{i}^{\left(\pi ,\pi^{\prime }\right)}={\hat{\lambda }}_{i}\left(X^{\pi },t^{\pi^{\prime }}\right)$ and ${\hat{\sigma }}_{h}^{2(\pi ,\pi^{\prime })}$ = ${\hat{\sigma }}_{h}^{2}\left(X^{\pi },t^{\pi^{\prime }}\right)$. Note that the possible number of random permutation is considerably increased when permuting both X and t independently.

It turns out that the distribution of the test statistic $fh({\hat{\lambda }}_{1},{\hat{\lambda }}_{2})$ differs in the general model (1) from its permutation distribution, and a valid level α test can not be achieved in this setup. Therefore, we consider the distribution of the test statistic $T_{\left(h\right)}$ defined in (15) and of the studentized quantity

(19) $$\begin{array}{c} T_{\left(h\right)}^{\left(\pi ,\pi^{\prime }\right)}=f\frac{h\left({\hat{\lambda }}_{1}^{\left(\pi ,\pi^{\prime }\right)},{\hat{\lambda }}_{2}^{\left(\pi ,\pi^{\prime }\right)}\right)}{{\hat{\sigma }}_{h}^{\left(\pi ,\pi^{\prime }\right)}}. \end{array}$$

The conditional limiting distribution of $T_{\left(h\right)}^{\left(\pi ,\pi^{\prime }\right)}$ given the data X will be derived in the next theorem.

Theorem 5.1 Let $T_{\left(h\right)}^{\left(\pi ,\pi^{\prime }\right)}$ as given in (19) and denote by Φ(x) the standard normal distribution function. If $\sigma_{L}^{2}>0$, then we have convergence under the null as well as under the alternative with

$$\begin{array}{c} \underset{x\in R}{\sup}\left|P\left(T_{\left(L\right)}^{\left(\pi ,\pi^{\prime }\right)}\leq x\right)-\Phi \left(x\right)\right|\overset{P}{\to }0. \end{array}$$

Theorem 5.1 states that the limiting standard normal distribution of $T_{\left(L\right)}^{\left(\pi ,\pi^{\prime }\right)}$ does not depend on the distribution of the data, particularly, it is achieved for arbitrary $h\left(\lambda_{1},\lambda_{2}\right)=\theta_{0}$, that is it even holds under the alternative.

Let $\psi_{f}^{\left(\pi ,\pi^{\prime }\right)}=1\left\{T_{\left(h\right)}\leq z_{\alpha /2}^{\left(\pi .\pi^{\prime }\right)}\right\}+1\left\{T_{\left(h\right)}\geq z_{1-\alpha /2}^{\left(\pi ,\pi^{\prime }\right)}\right\}$, where $z_{\alpha /2}^{\left(\pi ,\pi^{\prime }\right)}$ denotes the α/2‐quantile from the studentized permutation distribution of $T_{\left(L\right)}$. In the next theorem, we will show that both the conditional and unconditional tests are asymptotically equivalent, which means, that both tests have, asymptotically, the same power to detect certain alternatives.

Theorem 5.2 Suppose that the assumptions of Theorem 5.1 are fulfilled.

1. Under the null hypothesis $H_{0}:h\left(\lambda_{1},\lambda_{2}\right)=0$, the studentized permutation test $\psi_{f}^{\left(\pi ,\pi^{\prime }\right)}$ is asymptotically exact at α level of significance, that is $E(\psi_{f}^{\left(\pi ,\pi^{\prime }\right)})\to \alpha$, and asymptotically equivalent to $\psi_{f}$, that is
  $$\begin{array}{c} E\left(\left|\psi_{f}^{\left(\pi ,\pi^{\prime }\right)}-\psi_{f}\right|\right)\to 0,\;f\to \infty . \end{array}$$
2. The permutation test $\psi_{f}^{\left(\pi ,\pi^{\prime }\right)}$ is consistent, that is we have convergence
  $$\begin{array}{c} E\left(\psi_{f}^{\left(\pi ,\pi^{\prime }\right)}\right)\to \alpha 1\left\{h\left(\lambda_{1},\lambda_{2}\right)=0\right\}+1\left\{h\left(\lambda_{1},\lambda_{2}\right)\neq 0\right\},f\to \infty . \end{array}$$

In particular, Theorem 5.1 states that the distributions of the pivotal quantity $T_{\left(h\right)}$ and of the studentized permutation statistic $T_{\left(h\right)}^{\left(\pi ,\pi^{\prime }\right)}$ asymptotically coincide. Under the assumptions of Theorem 5.1, approximate (1−α)‐confidence intervals for θ can be obtained from

(20) $$\begin{array}{c} P\left(\theta \in \left[h\left({\hat{\lambda }}_{1},{\hat{\lambda }}_{2}\right)-\frac{z_{1-\alpha /2}^{\left(\pi ,\pi^{\prime }\right)}}{f}{\hat{\sigma }}_{h},h\left({\hat{\lambda }}_{1},{\hat{\lambda }}_{2}\right)-\frac{z_{\alpha /2}^{\left(\pi ,\pi^{\prime }\right)}}{f}{\hat{\sigma }}_{h}\right]\right)\to 1-\alpha . \end{array}$$

### 5.2 Simulation results

In a negative binomial‐$NB(t_{ik}\lambda_{i},\phi_{i})$‐model we investigate the empirical control of the preassigned type‐1 error rate at the usual two‐sided significance level α=5% of the statistic $T_{\left(L\right)}$ in (18) using the standard normal approximation as given in (15), and the permutation test using the quantiles of the conditional distribution of $T_{\left(h\right)}^{\left(\pi ,\pi^{\prime }\right)}$ in (19) as critical values. As a further competing procedure, we estimate the variances $\sigma_{i}^{2}$ using maximum likelihood methods. In this $NB(t_{ik}\lambda_{i},\phi_{i})$‐model the variance $\sigma_{i}^{2}$ is given by the weighted sequence of the quantities $t_{ik}\lambda_{i}+t_{ik}^{2}\lambda_{i}^{2}\phi_{i}$, respectively. An intuitive plug‐in estimation approach is achieved by replacing the unknown parameter $\lambda_{i}$ by ${\hat{\lambda }}_{i}$ from above and $\phi_{i}$ by a consistent maximum‐likelihood estimator (ML) ${\hat{\phi }}_{i}$, for example by using

(21) $$\begin{array}{ccc} {\ddot{\sigma }}_{i}^{2} & = & \frac{1}{T_{i}^{2}}\sum_{k=1}^{n_{i}}\left\{t_{ik}{\hat{\lambda }}_{i}+t_{ik}^{2}{\hat{\lambda }}_{i}^{2}{\hat{\phi }}_{i}\right\}, \end{array}$$

see, for example Schneider, Schmidli, and Friede (2013). This estimation approach, however, has the disadvantage that neither ${\hat{\lambda }}_{i}^{2}$ nor ${\hat{\phi }}_{i}$ are unbiased estimators of $\lambda_{i}^{2}$ or $\phi_{i}$, respectively, resulting in biased variance estimators. The variance estimators ${\hat{\sigma }}_{i}^{2}$ used in $T_{\left(L\right)}$ are finally replaced by ${\ddot{\sigma }}_{i}^{2}$, and the corresponding Wald‐statistic, which is asymptotically equivalent to the Likelihood‐ratio test, denoted by LRT.

#### 5.2.1 Type‐1 error rate simulations

We explore the behavior of the test statistics for smaller and larger effect rates λ_1_ and λ_2_ ∈{1.5,10} as well as smaller and larger overdispersion parameters ϕ_1_ and ϕ_2_ ∈{0.3,0.5,3,5}.

All simulation designs are motivated by the examples presented in Section 6. A major assessment criterion for the accuracy of the procedures is their behavior when increasing sample sizes are combined with increasing variance parameter constellations (positive pairing) or with decreasing variances (negative pairing). We investigate balanced situations with sample size vector $n_{1}={\left(n_{1},n_{2}\right)}^{\prime }=\left(7,7\right)$ and unbalanced situations with sample size vector $n_{2}=\left(n_{1},n_{2}\right)={\left(7,15\right)}^{\prime }$. The sample sizes are increased by adding a constant *m* to the components of the vectors $n_{1}$ or $n_{2}$, respectively. The different simulation settings are displayed in Table 1. Each simulation setting $n=n_{s}\left(m\right)={\left(n_{1}+m,n_{2}+m\right)}^{\prime }$ represents a different design with an increasing sample size *m*, where s=1,2, see Table 1.

**Table 1:** Simulated designs, where m∈{0,5,10,20,25} and $n_{1}={\left(7,7\right)}^{\prime }$, $n_{2}={\left(7,15\right)}^{\prime }$

Setting	$\lambda_{1}=\lambda_{2}$	Sizes	Overdisp.	Interpretation
1	1.5	$n=n_{1}+m$	$\phi =\phi_{1}$	Balanced/equal overdispersion
2	1.5	$n=n_{2}+m$	$\phi =\phi_{1}$	Unbalanced/equal overdispersion
3	1.5	$n=n_{1}+m$	$\phi =\phi_{2}$	Balanced/unequal overdispersion
4	1.5	$n=n_{2}+m$	$\phi =\phi_{2}$	Unbalanced/unequal overdispersion (positive pairing)
5	1.5	$n=n_{2}+m$	$\phi =\phi_{3}$	Unbalanced/unequal overdispersion (negative pairing)
6	10	$n=n_{1}+m$	$\phi =\phi_{4}$	Balanced/equal overdispersion
7	10	$n=n_{2}+m$	$\phi =\phi_{4}$	Unbalanced/equal overdispersion
8	10	$n=n_{1}+m$	$\phi =\phi_{5}$	Balanced/unequal overdispersion
9	10	$n=n_{2}+m$	$\phi =\phi_{5}$	Unbalanced/unequal overdispersion (positive pairing)
10	10	$n=n_{2}+m$	$\phi =\phi_{6}$	Unbalanced/unequal overdispersion (negative pairing)

Here $\phi_{1}={\left(\phi_{1},\phi_{2}\right)}^{\prime }={\left(0.3,0.3\right)}^{\prime }$, $\phi_{2}=\left(\phi_{1},\phi_{2}\right)={\left(0.3,0.5\right)}^{\prime }$, $\phi_{3}=\left(\phi_{1},\phi_{2}\right)={\left(0.5,0.3\right)}^{\prime }$, $\phi_{4}={\left(\phi_{1},\phi_{2}\right)}^{\prime }={\left(3,3\right)}^{\prime }$, $\phi_{5}=\left(\phi_{1},\phi_{2}\right)={\left(3,5\right)}^{\prime }$, and $\phi_{6}=\left(\phi_{1},\phi_{2}\right)={\left(5,3\right)}^{\prime }$ denote vectors of overdispersion parameters and $n_{i}+m$ means that every component of $n_{i}$, that is each group size, is increased by *m*.

**Figure 1:**
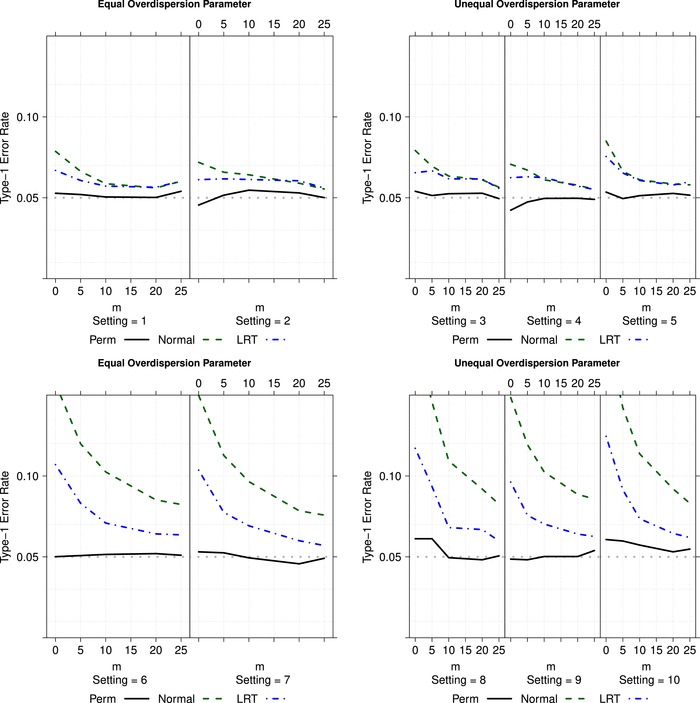
Type‐I error level (α = 5%) simulation results (y‐axis) of the statistics $T_{\left(L\right)}$ in (18), permutation test $T_{\left(h\right)}^{\left(\pi ,\pi^{\prime }\right)}$ in (19) and ML‐based statistics for different distributions, sample size increments m∈{0,5,10,15,20,25} (x‐axis), where $t_{ik}$ denote the realizations from $T_{ik}∼U\left(1,2\right)$. The simulation settings are described in Table 1

Data were generated from $X_{ik}∼NB\left(t_{ik}\lambda_{i},\phi_{i}\right)$, where $t_{ik}$ denotes the realization from a uniformly distributed random variable $T_{ik}∼U\left(1,2\right)$, respectively. For each simulation setting, the same generated follow‐up times $t_{ik}$ were used for the $n_{sim}=10,000$ simulation runs, but they were newly generated for each design. The number of random permutations was set to $n_{perm}=10,000$. The simulated type‐1 error rates for a significance level α=5% assuming uniformly distributed follow‐up times are displayed in Figure 1.

It turns out that in case of small effect rates ($\lambda_{1}=\lambda_{2}=1.5$) and small overdispersion parameters the statistics $T_{\left(L\right)}$ based on the normal approximation as well as the LRT statistics based on ML tend to be slightly liberal. It can be readily seen from Figure 1 that the permutation tests control the type‐1 error rate best, even for extremely small sample sizes. In case of larger effect rates and overdispersion parameters the distribution of the data is much more skewed. In these situations the procedures $T_{\left(L\right)}$ based on the normal approximation and ML tend to considerably overreject the null hypothesis $H_{0}^{\left(L\right)}$. Remarkably, the estimated type‐1 error rates are even larger than 20% and 10%, respectively in Settings 6–10 (see Figure 1). In comparison, the permutation technique greatly improves the finite sample performance of all asymptotic procedures, and is therefore recommended in practical applications.

In order to investigate the impact of the underlying distributions of the follow‐up times, we resimulate the same designs with exponentially distributed follow‐up times $T_{ik}∼Exp\left(2\right)+1$. The results are displayed in the supplementary material. It can be seen that the shape of the underlying follow‐up times distributions slightly affect the behavior of the statistics in all scenarios. This is intuitively clear, since the different follow‐up times particularly influence the variance of the effect estimators, and increase the variance with wider ranging follow‐up times or certain amount of skewness. Therefore, all procedures tend to be slightly more liberal when wide ranging follow‐up times and small sample sizes are apparent. This can be particularly seen by the permutation test. The liberality, disappears with increasing sample sizes.

#### 5.2.2 Power comparisons

The type‐1 error rate simulation results presented in Section 5.2.1 indicate a quite liberal behavior of the methods $T_{\left(L\right)}$ and ML‐based statistics under certain parameter constellations and small sample sizes. All methods tend to accurate conclusions with large sample sizes. The liberality of these methods increases the “power” of the methods to detect alternatives in small sample size settings. In an additional simulation study, not presented here, it turned out, that with large sample sizes, that is when all competing methods are accurate, their powers are all very similar.

#### 5.2.3 Simulated coverage rates of the confidence intervals

**Figure 2:**
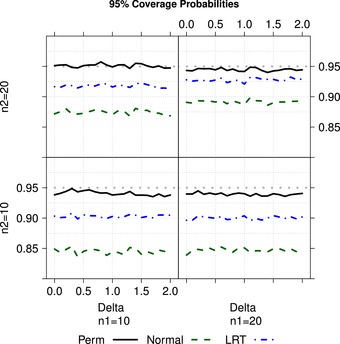
Empirical coverage probabilities of nominal 95% confidence intervals of the corresponding confidence intervals given in (16), permutation‐ based confidence intervals given in (20) and ML‐based LRT statistics for different distributions and rate increments δ∈{0,0.1,…,2} (x‐axis) and unequal overdispersion parameters ($\phi_{1}=3,\phi_{2}=5$), where $t_{ik}$ denote the realizations from $T_{ik}∼U\left(1,2\right)$

Next we investigate the empirical coverage probabilities of the corresponding confidence intervals. Data were generated by $X_{1k}∼NB\left(\lambda_{1}t_{1k},\phi_{1}\right),k=1,\ldots ,n_{1}$ and $X_{2k}∼NB\left(\lambda_{1}\left(1+\delta \right)t_{2k},\phi_{2}\right)$ for varying δ∈{0,0.1,0.2,…,2}, $n_{1},n_{2}\in \left\{10,20\right\}$, and different overdispersion parameters. For illustration purposes, we only display the results using uniformly distributed follow‐up times, different overdispersion parameters $\phi_{1}=3$ and $\phi_{2}=5$ and rate $\lambda_{1}=10$. The results are displayed in Figure 2. It is readily seen that the competing procedures tend to be rather liberal, while the empirical coverage probabilities of the permutation‐based confidence intervals are closer to the nominal level of 95%. The quality of the approximation depends on sample sizes and the actual levels of heteroscedasticity across the groups and their allocations. If the larger sample has a smaller variance than the smaller sample ($n_{1}=20,n_{2}=10$), the confidence intervals tend to be slightly liberal for small samples. However, this issue vanishes with increasing sample sizes.

#### 5.2.4 Simulation results for general metric data

As mentioned in the Introduction and in the description of model (1), data is not required to be count data and thus, numerical investigations of the behavior of the studentized permutation test are intriguing. We therefore investigate the empirical control of the type‐1 error rate of the studentized permutation test $T_{\left(L\right)}^{\left(\pi ,\pi^{\prime }\right)}$ in completely heteroscedastic designs with metric data following exponential or χ^2^‐distributions. The method will be compared with $T_{\left(L\right)}$ using the standard normal approximation. Exponentially distributed variables were generated by $X_{ik}∼Exp\left(t_{ik}\cdot 1/2\right)$, $i=1,2,k=1,\ldots ,n_{i}$, and χ^2^‐variables were generated by $X_{ik}∼\chi_{t_{ik}}^{2}$, respectively.

The results are displayed in Table 2 and show that the studentized permutation approach controls the nominal type‐1 error rate very well and greatly improves the standard normal approximation.

**Table 2:** Type‐I error level (α = 5%) simulation results of the statistics $T_{\left(L\right)}$ in (18) and the permutation test $T_{\left(L\right)}^{\left(\pi ,\pi^{\prime }\right)}$ in (19) using χ^2^‐square and exponentially distributed data in different designs, where $t_{ik}$ denote the realizations from $T_{ik}∼U\left(1,2\right)$

		$X_{ik}∼\chi_{t_{ik}}^{2}$		$X_{ik}∼Exp\left(t_{ik}\cdot 1/2\right)$	
*n*_1_	*n*_2_	$T_{\left(L\right)}^{\left(\pi ,\pi^{\prime }\right)}$	$T_{\left(L\right)}$	$T_{\left(L\right)}^{\left(\pi ,\pi^{\prime }\right)}$	$T_{\left(L\right)}$
7	7	0.0567	0.1168	0.0440	0.0937
7	15	0.0479	0.1063	0.0373	0.0884
12	12	0.0521	0.0862	0.0500	0.0807
12	20	0.0473	0.0825	0.0364	0.0644
17	17	0.0498	0.0757	0.0355	0.0565
17	25	0.0521	0.0769	0.0540	0.0822
27	27	0.0535	0.0694	0.0854	0.1058
27	35	0.0522	0.0684	0.0454	0.0618
32	32	0.0544	0.0698	0.0469	0.0575
32	40	0.0494	0.0634	0.0526	0.0623

## 6 TWO ILLUSTRATIVE EXAMPLES

Pediatric MS with disease onset under the age of 16 is uncommon and qualifies as a rare disease. Differences in clinical presentation before and after puberty have been reported (Huppke et al., 2014). Randomized controlled trials in pediatric MS have been very rare (Unkel et al., 2016), but are becomming more common now (Rose & Müller, 2016). We consider a randomized controlled trial assessing efficacy and safety of interferon beta‐1a compared to no treatment in pediatric MS reported by Pakdaman, Fallah, Sahraian, Pakdaman, and Meysamie (2006). In this trial, 16 patients were randomized to verum or control. Relapse rates and new T2 lesions were both considered as endpoints. The estimated rates and overdispersion parameters are given in Table 3. As a second example, we consider the Acyclovir trial reported by Lycke et al. (1996). In this experiment, Acyclovir treatment was used in a randomized, double‐blind, placebo‐controlled clinical trial with parallel groups to test the hypothesis that herpes virus infections are involved in the pathogenesis of MS. In total, N=60 adult patients were recruited, whereas $n_{1}=n_{2}=30$ were randomized to placebo or active treatment, respectively. The data (relapse counts) can be found in Figure 1 in the original publication (Lycke et al., 1996). As a secondary analysis of this trial, the relapse counts from patients that showed a progressive course during the trial were excluded from the statistical analysis. In this situation, patients have different follow‐up times and estimators must be weighted accordingly.

**Table 3:** Estimated rates and overdispersion parameters (Variance / Mean Ratio) for the two example studies

Endpoint	Group	Estimated rate ${\hat{\lambda }}_{i}$	Sample variance	Estimated overdispersion
Pediatric MS trial (*N*=16)				
T2 lesions	Control	11.875	13.268	1.117
	Active	10.625	16.839	1.585
Relapses	Control	4.5	6.571	1.460
	Active	2.375	0.268	0.113
Acyclovir trial (*N*=60)				
Relapses	Control	3.133	6.602	2.107
	ACYC	2.067	3.030	1.466
Acyclovir trial (*N*=60; Secondary analysis)				
Relapses	Control	3.205	6.602	2.060
	ACYC	2.118	3.172	1.498

The estimated rates and overdispersions being defined as variance‐to‐mean ratios are given in Table 3. It can be readily seen from Table 3 that the overdispersion parameters seem to differ between the treatment groups, and even underdispersed counts are apparent. The effect of the different overdispersion parameters on the behavior of the statistical methods has been analyzed in detail in extensive simulation studies in Section 5.2.

Both motivating examples discussed above used over‐ and underdispersed counts as outcomes. Here, we present the results based on standard methods including normal approximation and maximum‐likelihood as well as the new developed methods. The test statistic being used is given by

(22) $$\begin{array}{c} T_{NB}=\frac{\log\left({\hat{\lambda }}_{1}/{\hat{\lambda }}_{2}\right)}{\sqrt{\frac{{\ddot{\sigma }}_{1}^{2(c,P)}}{{\hat{\lambda }}_{1}^{2(c)}}+\frac{{\ddot{\sigma }}_{2}^{2(c,P)}}{{\hat{\lambda }}_{2}^{2(c)}}}}, \end{array}$$

where

$$\begin{array}{ccc} {\ddot{\sigma }}_{i}^{2} & = & \frac{1}{T_{i}^{2}}\sum_{k=1}^{n_{i}}\left\{t_{ik}{\hat{\lambda }}_{i}+t_{ik}^{2}{\hat{\lambda }}_{i}^{2}\hat{\phi }\right\}, \end{array}$$

denotes the estimated variance of the effect estimator using a MLE estimator of the overdispersion parameter ϕ, which is assumed to be identical across both treatment groups.

As competing methods, we also analyze the data using both a Negative Binomial Regression‐ and Poisson Regression using *SAS PROC GENMOD*.

Thus, the illustrative examples include constant as well as varying follow‐up times, and even the analyses with constant follow‐up times still presents a challenge since the sample sizes are with 16 and 60 very and moderately small, and the overdispersion is fairly pronounced, in particular for the MRI lesion counts and relapses. The effect estimates, standard errors, test statistics, *P*‐values as well as 95%‐confidence intervals are displayed in Table 4.

**Table 4:** Statistical analysis of the examples using $h\left(\lambda_{1},\lambda_{2}\right)=log\left(\lambda_{1}/\lambda_{2}\right)$: Approximate method, Effect ($log({\hat{\lambda }}_{1}/{\hat{\lambda }}_{2})$), Standard Error (SE), Test Statistic (= Effect / SE), and 95% confidence intervals

Method	Effect	SE	Statistic	*P*‐value	95% CI
T2 lesions					
Normal (15)	0.111	0.174	0.638	0.524	(−0.231; 0.453)
LRT (21)	0.111	0.162	0.686	0.493	(−0.207; 0.429)
LRT.Pool (22)	0.111	0.161	0.691	0.489	(−0.204; 0.427)
Perm (19)	0.111	0.174	0.638	0.545	(−0.269; 0.510)
NB‐Reg	0.111	0.161	0.691	0.489	(−0.204; 0.428)
Pois‐Reg	0.111	0.149	0.745	0.456	(−0.181; 0.405)
Relapses					
Normal (15)	0.639	0.216	2.964	0.003	(0.216; 1.062)
LRT (21)	0.639	0.302	2.116	0.034	(0.047; 1.231)
LRT.Pool (22)	0.639	0.284	2.254	0.024	(0.083; 1.195)
Perm (19)	0.639	0.216	2.964	0.026	(0.116; 1.162)
NB‐Reg	0.639	0.284	2.254	0.024	(0.096; 1.215)
Pois‐Reg	0.639	0.284	2.254	0.024	(0.096; 1.215)
Acyclovir relapses					
Normal (15)	0.416	0.215	1.939	0.052	(−0.004; 0.837)
LRT (21)	0.416	0.228	1.824	0.068	(−0.031; 0.863)
LRT.Pool (22)	0.416	0.231	1.805	0.071	(−0.036; 0.868)
Perm (19)	0.416	0.215	1.939	0.054	(−0.007; 0.842)
NB‐Reg	0.416	0.231	1.805	0.071	(−0.035; 0.870)
Pois‐Reg	0.416	0.164	2.544	0.011	(0.098; 0.741)
Acyclovir relapses (Secondary analysis)					
Normal (15)	0.414	0.218	1.904	0.057	(−0.012; 0.841)
LRT (21)	0.414	0.230	1.798	0.072	(−0.037; 0.866)
LRT.Pool (22)	0.414	0.233	1.781	0.075	(−0.076; 0.845)
Perm (19)	0.414	0.218	1.904	0.062	(−0.022; 0.845)
NB‐Reg	0.415	0.233	1.780	0.075	(−0.040; 0.874)
Pois‐Reg	0.422	0.165	2.553	0.011	(0.101; 0.750)

It can be readily seen from Table 4, that the estimated standard errors of the effect estimates for the T2 lesions are likely, and therefore all methods results in the same conclusion. Only the estimated standard error being computed via a Poisson‐Regression tends to be smaller. This occurs because the Poisson‐Regression sets the overdispersion to be zero, by default. A significant effect at 5% level can not be detected with any method (*P* > 0.05). The relapse rates are significantly different at 5%‐level of significance. It can be seen, however, that the estimates of the standard errors significantly differ from the moment‐based unbiased variance estimators (SE = 0.216 vs. SE = 0.302 using ML). Therefore, the *P*‐values based on ML estimates are larger than using the moments‐based estimator and standard normal distribution (*P* = 0.003 vs. *P* = 0.034). However, since sample size is rather small, the permutation approach is the most robust method in this setup, and results in a *P*‐value of *P* = 0.026. Since both over‐ and underdispersed counts were observed, the ML.Pool, the negative binomial, and poisson regression are tend to provide identical results.

The results obtained for the Acyclovir trial, however, differ significantly. First, both treatment groups show a different overdispersion. Therefore, the SE obtained by a Poisson‐Regression is way smaller than with all other methods, and thus results in a significant treatment effect at 5% level of significance. Comparing the other estimation approaches it can be seen that the ML‐based estimation approaches (assuming negative binomial distribution) of the SE tend to be larger than the unbiased methods‐of‐moments based methods. The largest SE is estimated via ML.Pool (which is identical to a NB‐Regression). The estimated standard error based on the unbiased variance estimate is given by SE = 0.215. Therefore, the *P*‐values range from 0.052 through 0.071. Due to the moderate sample size of N=60, both the normal and permutation approximation tend to provide similar *P*‐values with P=0.052 and P=0.054, respectively. The secondary analysis of the the Acyclovir trial shows similar results to the above. This occurs because only the relapse counts from four of the 60 patients were excluded from the analysis. However, slightly different effect estimates coming from the Negative Binomial and Poisson Regression can be seen. This occurs, because in case of unequal follow‐up times the rates are estimated using maximum likelihood estimation methods, which are not identical to moment (mean‐based) methods.

## 7 DISCUSSION

In this paper, inference methods for testing hypotheses formulated in terms of the effect rates of overdispersed counts were developed without assuming a specific data distribution and/or different overdispersion parameters. They are based on the asymptotic properties of novel unbiased estimators of the count rates and their variances. In order to provide valid methods for small sample sizes, resampling methods have been derived. Although data is in general not exchangeable, following the ideas of Neuhaus (1993), Janssen (1997, 2005), and Chung and Romano (2013), studentized permutation techniques could be applied. Simulation studies indicate, however, that the procedures control the nominal level reasonably well even with $n_{i}\approx 5$.

Furthermore, in clinical trials, the computation of confidence intervals for the treatment effects is important, following the ICH E9 guideline for randomized clinical trials: “*Estimates of treatments shall be accompanied by confidence intervals, whenever possible&* (ICH E9 Guideline 1998, chap. 5.5, p. 25). For instance, Saha (2013) investigates different methods for the computation of confidence intervals for the mean difference in the analysis of overdispersed count data (assuming constant follow‐up times $t_{ik}$). In this paper, these procedures were generalized for possibly time‐varying and overdispersed count data and equipped with the studentized permutation approach. Extensive simulation studies show that the new methods improve the existing methods in terms of coverage probability and type‐*I*‐error rate control. Furthermore, we only considered one possible unbiased estimator of the rates $\lambda_{i}$ by ${\hat{\lambda }}_{i}=\frac{1}{T_{i}}\sum_{k=1}^{n_{i}}X_{ik}$, which is known as a weighted mean estimator. Another unbiased estimator is given by the unweighted mean ${\hat{\lambda }}_{i}^{\left(u\right)}=\frac{1}{n_{i}}\sum_{k=1}^{n_{i}}\frac{X_{ik}}{t_{ik}}$, or least‐square based estimators ${\hat{\lambda }}_{i}={\left(t_{i}^{\prime }t_{i}\right)}^{-1}t_{i}^{\prime }X_{i}$, where $t_{i}={\left(t_{i1}.\ldots ,t_{in_{i}}\right)}^{\prime }$ and $X_{i}={\left(X_{i1},\ldots ,X_{in_{i}}\right)}^{\prime }$ denote the vectors of follow‐up times and response per group *i*, respectively. Investigating and comparing those estimators and generalizations thereof is tempting and will be subject to future research.

In future investigations, the results shall be extended to more general models allowing for covariates (e.g. for baseline adjustment) and several samples. Furthermore, investigating the overlap of range‐preserving confidence intervals for the effects is an interesting attempt for making inferences (Noguchi & Marmolejo‐Ramos, 2016).

## CONFLICT OF INTEREST

The authors have declared no conflict of interest.

## Supporting information

Supporting Information
