# Supplementary material for: Semi‐parametric analysis of overdispersed count and metric data with varying follow‐up times: Asymptotic theory and small sample approximations
Source: Biom J. 2018 Dec 5;61(3):616–29. doi: 10.1002/bimj.201800027 (PMC6587510; doi:10.1002/bimj.201800027)
Supplement: Supplementary file 1 — Supporting Information [file BIMJ-61-616-s001.zip › README.pdf]

Software Documentation for Semi-parametric analysis of over-dispersed count and metric data with varying follow-up times: Asymptotic theory and small sample approximations

Frank Konietschke[1,*], Tim Friede[2] and Markus Pauly[3]

Software Developer: Frank Konietschke

[*] Corresponding author. E-mail: *fxk141230@utdallas.edu*
[1] Department of Mathematical Sciences, University of Texas, Dallas, USA
[2] Department of Medical Statistics, University Medical Center Göttingen, Germany
[3] Institute of Statistics, University of Ulm, Germany

In this document supporting software is explained. The algorithms can be used for simulation studies, data evaluations and reproduction of the results obtained in the paper.

1 Simulation results and reproducing Figure 1

1. Open the source code of *simulations.R* and paste the code in *R*. The function

   ```
   CIperm(n1,n2,mu1,delta,phi1,phi2,nsim,nperm,Setting,nadd){...}
   ```

   has the following arguments

   - n1: Sample size in group 1
   - n2: Sample size in group 2
   - mu1: True rate parameter of the counts in group 1
   - delta: Shifting parameter to set the value of the count rate in group 2 (0 under the hypothesis)
   - phi1: Overdispersion parameter in group 1
   - phi2: Overdispersion parameter in group 2
   - nsim: Number of simulations
   - nperm: Number of permutations
   - Setting: Helping variable for graphical representation
   - nadd: add-on sample sizes (indicated as m in Figure 1)

2. Open the file *Figure1.R* and paste the code in the *R* console. The four graphics will be displayed upon the results of the simulation study at the beginning of the file. Intermediate results will be printed on the *R* console. They are also provided in the *Intermediate Results* folder.

2 Figure 2

The empirical coverage probabilities can be computed using the *CIperm* function described above.

1. Open the file *simulations.R* and copy paste the code in the *R* console.

2. Open the file *Figure2.R* and copy paste the code in the *R* console. The graphic Figure 2 will be displayed upon the simulation results. Intermediate results will be printed on the *R* console. They are also provided in the *Intermediate Results* folder.

3 Reproducing the results in Table 2

In Table 2, other distributions than the negative binomial distribution are simulated. The tests can be computed as follows:

1. Open the file *simulations Table2.R*. The function

```
CIperm(n1,n2,nsim,nperm,Setting,nadd, Distribution){...}
```

has the following arguments:

- n1: Sample size in group 1
- n2: Sample size in group 2
- nsim: Number of simulations
- nperm: Number of permutations
- Setting: Helping variable for graphical representation
- nadd: add-on sample sizes (indicated as m in Figure 1)
- Distribution: Character string specifying the distribution (χ^2, exponential, ...)

2. Copy and paste the code in the *R* console. Table 2 will be displayed upon the simulation results. Intermediate results will be printed on the *R console. They are also provided in the Intermediate Results folder.*

4 Tables 3 and 4

The data evaluation results can be reproduced using the function *CIperm* within the *R*-function *case_study.R*. The function has the following arguments

```
CIperm(x,y,t1,t2,nperm)
```

- x: Data vector of data in group 1
- y: Data vector of data in group 2
- t1: Vector of corresponding follow-up times of the data x
- t2: Vector of corresponding follow-up times of the data y
- : nperm: Number of permutations

The data can be analyzed as follows:

1. Open the file *case_study.R* and copy paste the code in the *R* console. The results provided in Table 3 and 4 will be displayed in *R*. Note: Since the data sets are so small, they are provided below the code.
